# Mimicking the Martian Hydrological Cycle: A Set-Up to Introduce Liquid Water in Vacuum

**DOI:** 10.3390/s20216150

**Published:** 2020-10-29

**Authors:** Jesús Manuel Sobrado

**Affiliations:** Centro de Astrobiología (INTA-CSIC), Torrejón de Ardoz, 28850 Madrid, Spain; sobradovj@inta.es

**Keywords:** mars simulation, artificial atmosphere, water cycle, moss survival

## Abstract

Liquid water is well known as the life ingredient as a solvent. However, so far, it has only been found in liquid state on this planetary surface. The aim of this experiment and technological development was to test if a moss sample is capable of surviving in Martian conditions. We built a system that simulates the environmental conditions of the red planet including its hydrological cycle. This laboratory facility enables us to control the water cycle in its three phases through temperature, relative humidity, hydration, and pressure with a system that injects water droplets into a vacuum chamber. We successfully simulated the daytime and nighttime of Mars by recreating water condensation and created a layer of superficial ice that protects the sample against external radiation and minimizes the loss of humidity due to evaporation to maintain a moss sample in survival conditions in this extreme environment. We performed the simulations with the design and development of different tools that recreate Martian weather in the MARTE simulation chamber.

## 1. Introduction

There are similitudes and differences between Mars, the red planet, and Earth. One of the requirements for Earth-like life to emerge from a planet habitability perspective is the hydrological cycle. Pressure, temperature, and gas composition allow, for example, extreme microorganisms to live on Earth in different environments [1,2,3,4] where these variables are far from the average values.

**Figure 1:**
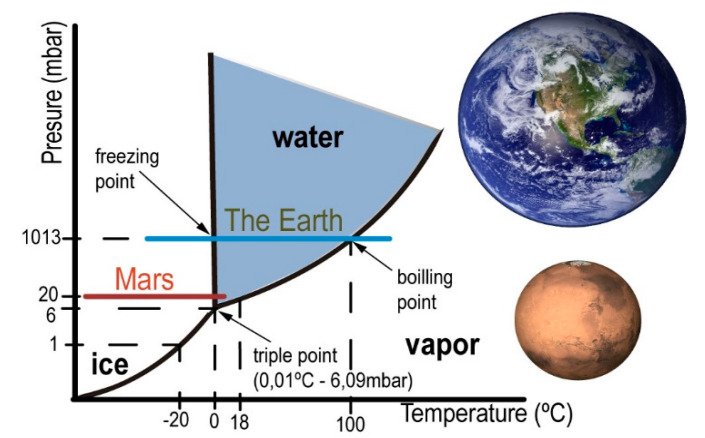
Water phase diagram [14]. Comparison of parameters between Mars and Earth.

Water is a prerequisite for life [5]. This constitutes the main astrobiology paradigm [6,7,8]. Water in the gaseous state is found mostly in the universe in the interstellar medium [9]. Water as ice abounds in the interior of some moons, such as Europe [10]; at the poles of rocky planets, such as Earth and Mars [11]; and in interplanetary objects, such as comets and asteroids [12]. In liquid state, water is located on the surface of the Earth and interior of satellites of the giant planets [13]. In principle, liquid water and vacuum are incompatible. Only under special conditions, liquid water can be introduced in a vacuum system. The water vapor pressure indicates that under certain conditions of pressure and temperature [14], it is possible for water to remain in liquid state (Figure 1).

Fortunately, Mars has water, as ice at the poles, as vapor in the atmosphere with a low percentage in comparison with the main gases (95% CO_2_, 2.7% N_2_; 1.6% Ar; 0.13% H_2_0; 0.08% CO) [15], and as liquid in subglacial layers [16,17]. Mars has seasons, diurnal and nocturnal variations of the main environmental variables [18] (temperature, pressure, radiation, composition of the atmosphere and humidity), which result in changes in the state of water in a short amount of time [19]. Under these conditions, ice–liquid and ice–mineral interfaces [20] are the appropriate means for extreme microorganisms to adapt in this hostile environment [21]. Some of the most relevant aspects from the point of view of liquid water on the surface of Mars are found in the hydrological cycle [22] and the daily humidity cycle that might influence hydration on Mars [23]. The hydrological cycle is related to the seasons, the ice of the polar caps, and the gas flow in the atmosphere [24].

The existence of water is essential for life to emerge. In planetary sciences, there have been developments of simulation systems that include water in habitability and geochemical studies of vacuum samples [25,26,27,28]. The last calibration of the Phoenix lander in relation to the relative humidity sensor [29,30] provides the means under very special circumstances to enable the existence of liquid water on the Martian surface [31]. According to this last calibration at the location of the Phoenix lander (near the North Pole), relative humidity values surpassing 35% have been recorded. In summer, a water vapor peak (relative humidity) appears due to the minimum CO_2_ ice coverage of the water ice formed in the bottom main part of the northern polar cap. Water vapor is transported to the equator from the polar caps of Mars [32,33]. This increase in water vapor, although it is a very small amount by volume of water due to the density of the atmosphere with an average pressure of ~(6–8) mbar [34], can condense on the cooler surfaces at dawn and occasionally at lower latitudes, especially at night [35], similar to what happens with dew on Earth. The average temperature in the air is 218 K (−55 °C), with a maximum of 276.3 K (3.15 °C) in the equator, up to 170 K (below −100 °C) in the polar cap [36]. Mars has a difference of up to 30 °C between the ground and the surface atmosphere [37]. On the other hand, the increase in diurnal temperature, the incidence of solar radiation on the surface, as well as the fluctuation and increase in pressure due to the sublimation of carbon dioxide ice, can allow that liquid water to appear as ice near the poles in short seasonal periods.

The regions near the poles of the red planet contain large amounts of water ice, both inside [16] and outside, giving rise to the existence of salts that produce a brine [38,39,40] of clathrates [41] and perchlorates [42,43]. On Mars, water ice and carbon dioxide are subject to climatic and seasonal and daily variations [44]. We know that an ice sheet protects against life-damaging ultraviolet radiation and is a good thermal insulator [45,46]. The main example on our planet is found in Antarctica [47], where micro algae composed mainly of cyanobacteria are capable of photo synthesizing [48,49]. In this setting, one of the places where water existence is possible in several states as well as the emergence of life is in locations favored by the hydration of the atmosphere.

The goal of this paper is to present an experimental set-up and method capable of recreating the appropriate environmental conditions to favor the metabolic activity of simple Earth-based organisms on the Martian surface [50]. We designed an observation in the laboratory similar to the reality on Mars that allows us to create a niche where it is possible to maintain a biological sample in this extreme environment. We simulated the sublimation, evaporation, melting, condensation, and freezing of water. In so doing, we provided our experimental system with the most appropriate environmental conditions to generate as completely as possible hydrological cycles in a vacuum system. Simulating the hydrological cycle of a planetary atmosphere in a vacuum system facilitates progress in the study of understanding the habitability of planetary objects, such as Mars.

We used the MARTE vacuum chamber of the CAB (“Centro de Astrobiología” or Astrobiology Center) [51] as a platform [52,53,54]. We used this vacuum chamber for its versatility in the integration of many environmental variables. In fact, the MARTE vacuum chamber was developed for testing the REMS (Rover Environment Monitoring Station) [55] of the MSL (Mars Science Laboratory) mission [56] on board the Curiosity rover.

We demonstrate in the laboratory that it is possible that a biological sample can survive in this environment. We used a moss sample (*sphagnum*) [57] from the glacial origin lagoon known as ‘los pájaros’ or The Birds in the Sierra de Guadarrama national park in Madrid, Spain (40°51.6126′ N; 3°56.8986′ W). The lagoon is at an altitude of 2170 m and, during the winter months, it has a layer of ice and snow on its surface.

Our simulation centers on 20 mbar pressure (Figure 1), which despite being a bit high for the red planet (with an average around 8 mbar), it is possible in some seasons and locations. The combination of low pressure with a temperature close to 0 °C allows H_2_O to pass from one state to another through different physical processes with small variations in energy.

In a vacuum chamber where the pumps operate without interruption to keep the pressure stable, it is complicated to create water droplets of condensation on biological samples without direct contact with cool surfaces.

We simulated condensation (hydration) by injecting liquid water directly in vacuum controlling the pressure and the temperature. We injected a layer of droplets that spread over the surface of the biological sample and drops that fall by decantation at the bottom to maintain the relative humidity in a range from 10% to 35% for the needs of the biological substrate. This technique based on (ALI) atomic layer injection [58] enables the temporal existence of liquid water in vacuum.

## 2. Design

**Figure 2:**
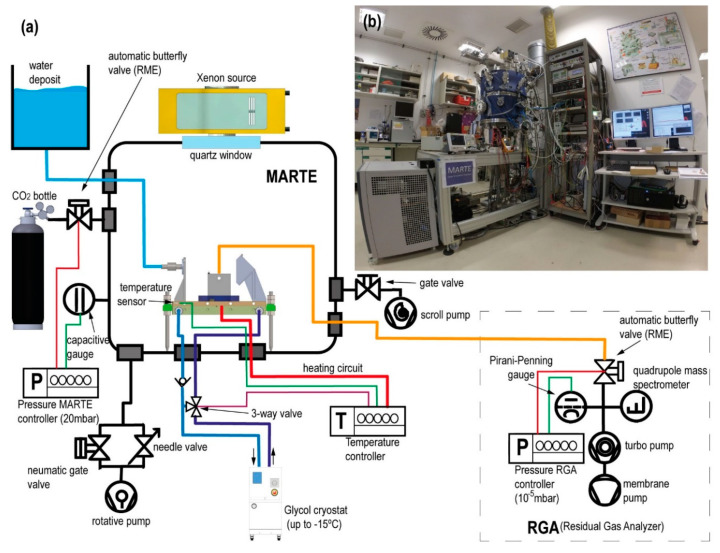
(**a**) Technical illustration of the MARTE simulation system (sample holder temperature control, gas composition, and pressure control). (**b**) Photograph of the MARTE simulation system.

In this section, we describe MARTE instrumentation, the water injection set-up in the vacuum, and the method employed in the simulation of the hydrological cycle and the control and monitoring of environmental variables, identifying the sensors and the operation mode. Figure 2 is a technical illustration and a photograph of the MARTE simulation chamber revealing the relevant equipment for liquid water simulation.

There are differences between the simulation systems and Mars (Table 1). We experimentally approximated the environmental variables in MARTE to maximize the residence time of liquid water in vacuum. In the simulation system, the initial composition and concentration of gases change due to the injections of liquid water in vacuum using the ALI pulse valve and for the outgassing of the biological sample (nitrogen, nitrogen dioxide, and water). Liquid water injection is measured in the vapor phase with a relative humidity sensor and with the mass spectrometer. The pressure increases necessary to achieve rapid and homogeneous changes in temperature (freezing the sample) are measured with gauges (capacitive, piezoresistive, and pirani) of different amplitude and response speeds. The radiation (ultraviolet, visible, and infrared) undergoes daytime and nighttime variations reproducible in the vacuum system by turning the radiation Xenon source on and off. The ice generated by absorption and for heat exchange with the sample holder produces an increase in the temperature and in the sample hydration, which are measured at the bottom of the Petri plate on which the sample is placed.

### 2.1. Water Control in Vacuum: Atomic Layer Injection

**Figure 3:**
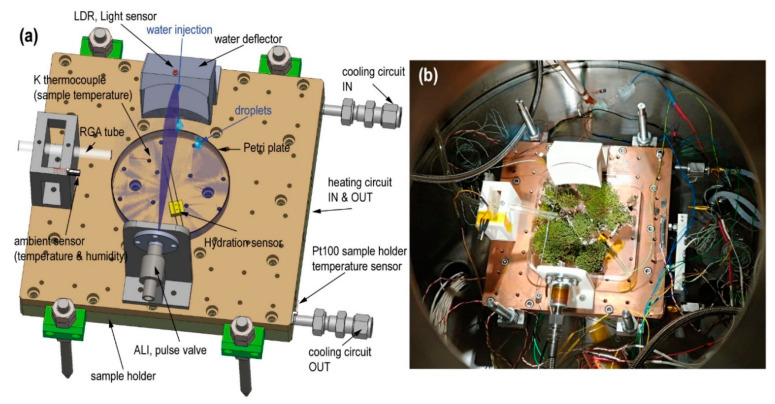
(**a**) Sample holder technical draw (top view) with all instrumentation for the water injection system. (**b**) Photograph (top view) of the experimental assembly with biological sample (moss).

In principle, liquids and vacuum are incompatible. The water vapor pressure on Mars along with the temperature produces either evaporation or instantaneous freezing. The main technological risk in vacuum is to achieve the existence of liquid water long enough [59] for photosynthesis to occur in an atmosphere of low-temperature carbon dioxide and exposure to solar radiation [60]. Thus, we adapted the ALI liquid injection system in the MARTE vacuum chamber (Figure 3).

**Figure 4:**
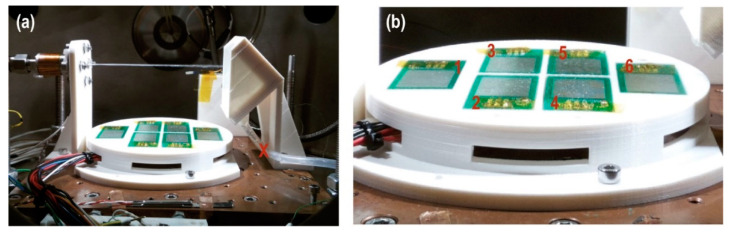
(**a**) Photograph taken during the injection pulse calibration on the “Dewsensor”. The red X shows the collector tube that collects the drops by decanting them to a reservoir located outside of the sample holder (Video S1). (**b**) Close-up of the droplet sensors. The red numbers identifying the sensors position.

Figure 3 shows the assembly of the injection system on the MARTE sample holder. The liquid injection system consists of a pulse valve mounted horizontally that injects liquid water over the deflector. The injection valve has a diameter of 1 mm. The water comes from the water tank that feeds the pulse valve, which is located outside MARTE (see Figure 2). The capacity of the external water tank is 15 L and is at a height of 1 m with respect to the valve. This height difference added to the pressure difference between the MARTE chamber (20 mbar) and the exterior (1 atmosphere) enables powerful water injections (see Figure 4a and Video S1) in short injection times, favoring water dispersion of the sample due to the impact with the deflector opposite the pulse valve. In vacuum, this is the chosen procedure to spray and to favor the droplets’ dispersal all over the surface. The water defector is fundamental in the system as it allows the jet caused by the injection to be broken in two ways: The first in droplets that are distributed on the surface of the sample (see Figure 4b), and the second by forming drops that fall by decantation on the Petri plate. In this way, the water is distributed over the surfaces exposed to the atmosphere and on the bottom of the Petri plate by dripping without breaking the sample.

#### 2.1.1. Injected Water Dispersion

**Figure 5:**
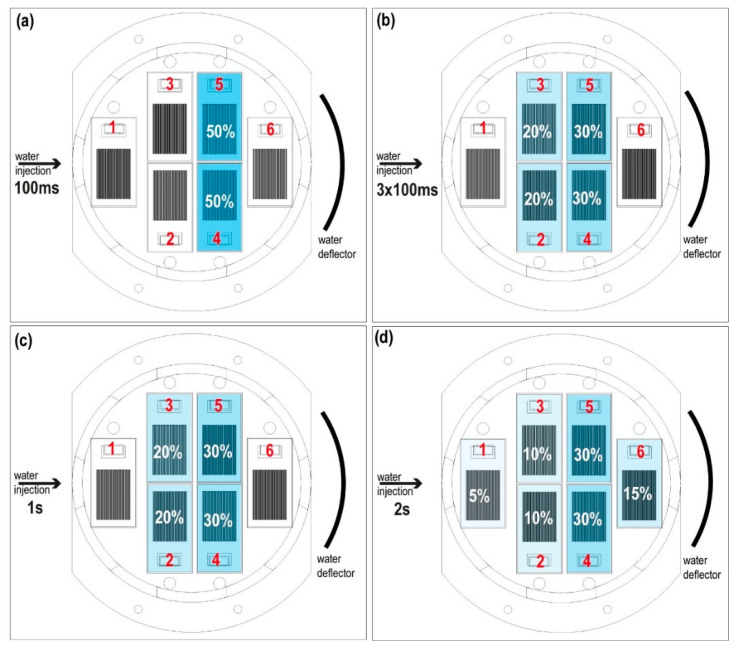
Top view of the “Dew sensor”. Position and coating (%) of water detectors for different injection times. The percentage and the color intensity show the total water droplets distribution. The red numbers indicate the drop water sensor position. (**a**) Pulse of 100 ms. (**b**) Combination of 2 series of 3 consecutive pulses of 100 ms with 1 s between series. (**c**) Pulse of 1 s. (**d**) Pulse of 2 s.

The dispersion and the coverage of the droplets is measured with the specific sensor “Dew sensor” (Figure 4 and Figure 5) formed by six detectors’ circuits. Each detector is mounted on a PCB (printed circuit board) forming an interlaced track voltage divider circuit of 80 microns placed 180 microns apart, thus there is no contact between them. The detection surface is 4.25 cm^2^ per board. Water droplets bounce off the deflector closing the circuit. The voltage decreases proportionally to the surface covered by water. The sensor detects water droplets greater than or equal to 440 microns in diameter. The coating and evaporation of the droplets on the detectors is related to the injection times. Thus, the dispersion (covered surface and preferred position) is observed and the evaporation rate of droplets on the sensors is measured with the lack voltage on the Dewsensor at 20 mbar of CO_2_ with an effective pumping speed S of $3.6\cdot 10^{-2}L\cdot s^{-1}$, which is the simulated operating range.

We verified (Figure 5a) that 100-ms pulses coated plates 4 and 5 with water droplets. In larger pulses, such as the combination of a sequence of three 100-ms pulses with one second between pulses, the sensor coating increased, reaching plates 2 and 3 (Figure 5b). The case of one pulse of 1 s is similar (Figure 5c). Only pulses with a duration of 2 s coated all plates (Figure 5d).

Finally, the average evaporation time of the droplets deposited on the detection plates for the 100 ms injection is about 10 s.

#### 2.1.2. Injected Water Mass

The injected water is the parameter that modifies the relative humidity and hydration of the sample (conductivity). We injected water for one second on a 10-cm-diameter Petri plate mounted on a load cell (force transducer) model PW4C3/300G-1 of HBM (Hottinger Brüel & Kjaer GmbH, Darmstadt, Germany) with a resolution of 0.05 g.

**Figure 6:**
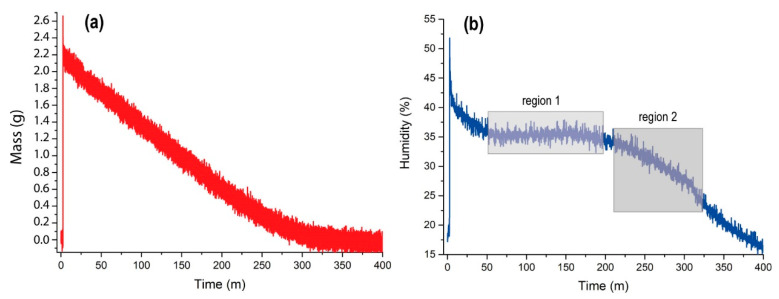
(**a**) Mass measurement of water injected and evaporated in 1 pulse of 1 s. (**b**) Relative humidity in the vacuum chamber during injection and evaporation. Region 1 shows the time in which the water sheet loses volume and the evaporation area and relative humidity remain constant. Region 2 shows the loss of volume and evaporation surface of the water layer decreasing the relative humidity.

Figure 6a shows the mass loss as a time function for an injection of one second from the valve at room temperature (22 °C) and 20 mbar pressure. At injection, a peak on the force transducer is produced by the water thrust. This value stabilizes once the water is distributed homogeneously across the surface of the Petri plate. The pressure control in MARTE at 20 mbar allows the entry of CO_2_ while maintaining a constant pumping speed of $3.6\cdot 10^{-2}L\cdot s^{-1}$. The water lost mass linearly and 325 min after the injection, it evaporated.

Under these conditions, 2.2 g of liquid water were injected, and evaporated in 325 min. The evaporation rate was 6.7 mg of water per minute.

In the case of the 100-ms water injection (Figure 5a), the droplets detected had an average evaporation time of 10 s, which corresponds to a water mass of 1.12 mg. Here, 99.5% of the 100-ms injection are drops that reach the bottom of the Petri plate by decantation and 0.5% are droplets on the surface.

Figure 6b shows the relative humidity measured by the ambient sensor (see Figure 3), which is formed by a combination between a Honeywell HIH-400 relative humidity sensor, and an RTD (resistance temperature device) temperature sensor type Pt1000 class A. At injection, there is an instantaneous evaporation that increases the relative humidity sharply and decreases the ambient temperature, resulting in short injection times (around 10 ms) and low-vacuum micro ice crystals [61]. In our case, once the water is distributed homogeneously through the Petri plate (the injection causes a sudden sharp rise in pressure), the humidity decreases until the pressure is stabilized at 20 mbar in MARTE. From the 50th minute onward, relative humidity is maintained at 35% (region 1 of Figure 6b) and begins to decay from the 200th minute to the 325th minute at 25%, which is when all the water from the Petri plate has evaporated (region 2 of Figure 6b). As of minute 325, the relative humidity decays exponentially to the threshold value of 20 mbar in the MARTE atmosphere.

As the water evaporated, the relative humidity stayed between 35% and 25%. The water evaporates, reducing the thickness of the layer of the 2.2 g water sheet, and preserving the effective evaporation from the surface as long as possible [62]. Therefore, it is possible to sustain optimal atmospheric relative humidity and hydration conditions by controlling water injection on a smooth pore-free surface. In the case of a sample with pores, the water penetrates the holes and the evaporation surface and the volume of water inside it is modified. The evaporation rate in a sample depends on the effective evaporation surface.

### 2.2. Hydration

Hydration (conductivity) is the parameter that enables us to ascertain the relative amount of liquid water within a biological sample. We used a homemade resistive conductivity meter sensor between the top of the Petri plate and the bottom of the biological sample in MARTE (Figure 3). Measurements were taken with a frequency of one second.

**Figure 7:**
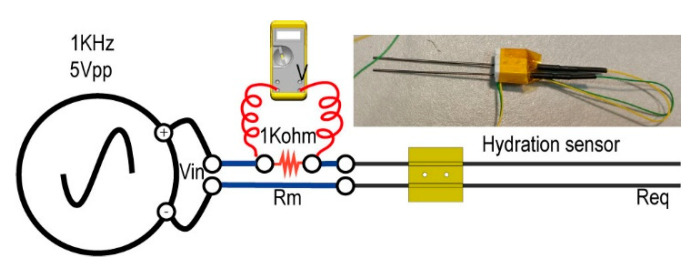
Technical illustration and photograph of the hydration measurement system. It consists of a function generator, a voltmeter, a resistance of 1 KOhm, and the hydration sensor.

Figure 7 shows the hydration sensor and the circuit to measure the equivalent sample resistance. The hydration sensor consists of two graphite bars of 0.7 mm in diameter and 60 mm in length, placed 7 mm apart.

The initial equivalent resistance corresponds to the value of the hydrated sample before it is introduced into the simulation chamber. Once in MARTE, the conductivity is modified under the effect of constant evaporation at 20 mbar. Water injection on the biological sample changes the hydration conditions, so it is possible to discern the hydration status of the sample in real time. In the case of dried or frozen samples, the equivalent resistance value is near a maximum of 1 KΩ.

### 2.3. Temperature

We measured the temperature in the three main elements inside MARTE: in the sample holder, in the atmosphere near the sample, and inside the biological sample at the bottom of the Petri plate (Figure 3a). The temperature on the surface of the MARTE copper sample holder is measured with an RTD sensor type Pt100 class A, located at the exit of the glycol circuit. Glycol and a 280 W resistor are used as heat exchange elements with the following temperature range from 253 to 283 K. The 3200 W Model K3 ATC compressor minimizes the thermal inertia of the sample holder and maintains a very stable temperature control with the Watlow F4S controller with PID (proportional integral differential) control, which acts on the valve that opens the glycol flow and on the electrical resistance of the sample holder (Figure 2). During the experiments, ambient temperature is monitored with a Pt1000 class A RTD, and the biological sample’s temperature is taken with a K-type thermocouple located at the bottom of the Petri plate.

### 2.4. Radiation

Mars has radiation characteristics that differ from Earth’s. Its tenuous atmosphere composed mainly of carbon dioxide, as well as its lower gravity and the absence of a global magnetic field means that there is a weaker and different filter than on Earth to prevent the arrival of energy particles and ultraviolet radiation [63]. Mars is more distant than Earth, 1.5 AU (astronomical units), thus its radiation values are different. UV (ultraviolet) radiation together with hydration are the two most influential variables in analyzing the subsequent survival of biological samples [64].

**Figure 8:**
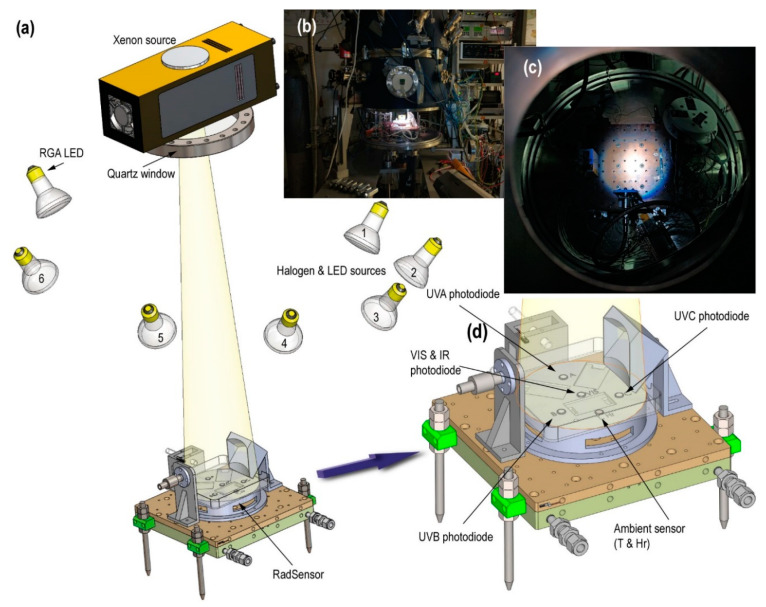
(**a**) Technical illustration of the radiation sources. (**b**) Photograph of MARTE with the lower cover open. The RadSensor is illuminated with the Xenon source. (**c**) Photograph of the sample holder illuminated from the top cover of MARTE. The light comes from the Xenon source. (**d**) Technical close-up of the RadSensor for UV radiation characterization.

Figure 8a illustrates the radiation sources in MARTE. We simulated the solar radiation with a Xenon source because it has a similar spectrum and irradiation levels can be scaled to the red planet’s surface. We used the Hamamatsu 150W L11033 lamp and a spectral range between 185 and 2000 nm mounted on the E7536 housing. The Xenon source is placed 80 cm from the surface, separated from the vacuum by an ISO 150 LF quartz window with a 90% optical transmission of UVA, and is far enough to minimize infrared radiation. Using the lens and the housing mirror, we adjusted the beam so that it covered the exposed surface of the biological samples in a homogeneous way (see Figure 8b,c). Finally, we used an RGB (red, green, blue)-type LED (light-emitting diode) lamp and halogen or LED sources in different positions that facilitate the contrast when taking pictures inside MARTE.

The radiation characterization is performed using a Pyranometer model CM6B of Kipp & Zonen, with a sensitivity of 13.71 µV/W·m^−2^ and a spectral range between 305 and 2800 nm at 80 cm from the Xenon source (MARTE chamber closed) at atmospheric pressure and room temperature. The irradiance of the Xenon source is 666.6 W/m^2^.

To monitor the UV radiation, we built a specific detection instrument, the «RadSensor» (Figure 8d). Made of ABS (acrylonitrile butadiene styrene), its surface contains three photodiodes of Ifw Optronics Gmbh assigned to each region of the UV spectrum. Additionally, there is an environmental sensor that combines the measurement of temperature and relative humidity in a TO-5 device model HIH-4602-C RTD by Honeywell. Table 2 shows the spectral range of the photodiodes and the irradiance of the UV radiation from the Xenon source.

### 2.5. Pressure and Atmosphere Control: Measuring Gas Composition

Pressure control on MARTE is performed in dynamic mode (the vacuum chamber is always pumping) with manual pumping conductance, through a fine adjustment valve and automatic variable gas flow of CO_2_ [65]. We regulate the conductance of the Pfeiffer DUO 20 vacuum pump with a needle valve. In this way, we optimize MARTE for the 20 mbar working pressure. Gas flow is minimal and variations in temperature and gas load due to water evaporation can be regulated more effectively. The pressure is measured with a Pfeiffer CMR 232 compensated temperature capacitive gauge with a range from 10^−2^ to 110 mbar and an APR 250 piezoresistive gauge with a range from 1 to 1000 mbar.

MARTE also has a Doc Edwards Scroll vacuum pump of 15 m^3^/h that is used for the absorption process in the interphase between the daytime and the nighttime along with the pump DUO 20 of 20 m^3^/h. In all cases, both pumps always have switched on the gas-ballast.

Humidity is measured in MARTE with the Honeywell HIH-400 sensor and all gases with the quadrupole mass spectrometer (Pfeiffer QMG 220) with a sensitivity (S) of 1.1A/mbar with the channeltron on and a range up to 200 uma operating through differential pumping (Figure 2). The measurement of the RGA (residual gas analyzer) enables us to ascertain the gases in the atmosphere inside MARTE.

The mass spectrometer connection with the MARTE chamber is regulated by a Pfeiffer RME 005 butterfly valve. This valve enables the gas entry from MARTE to the mass spectrometer, causing the equivalent of a leak of $5.95\cdot 10^{-4}\;\mathrm{mbar}\cdot L\cdot s^{-1}$ in the measurement chamber of the mass spectrometer to achieve a stable pressure of 10^−5^ mbar in the RGA equipment. With this base pressure, the channeltron (electron multiplier) can be connected, which maximizes the q/m signal for oxygen measurement (O_2_^+^ m/q = 32).

The connection between MARTE and the RGA is established through a corrugated tube in KF16 of 50-cm length to the MARTE chamber and a tube of 6 mm in internal diameter and 35 cm in length reaching the proximity of the sample, as seen in Figure 3.

Gas analysis is performed using a MID (multiple ion detector). The selected ions/masses correspond to oxygen (m/q = 16 and 32), nitrogen (m/q = 14 and 28), nitrogen dioxide (m/q = 30), water (m/q = 18), and carbon dioxide (m/q = 44), the main gases involved in the simulation of the hydrological cycle with the biological sample.

## 3. Performance

**Figure 9:**
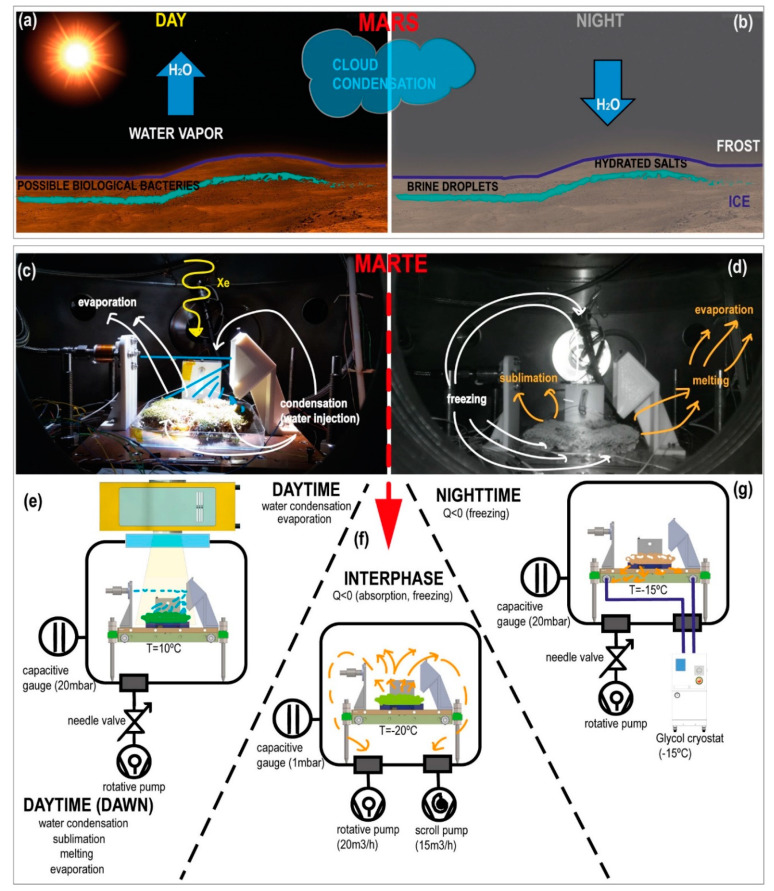
Pictures and technical illustrations of the hydrological cycle in the MARTE vacuum system and on the red planet. (**a**) Daytime in Mars. (**b**) Nighttime in Mars. (**c**) Daytime in MARTE. (**d**) Nighttime in MARTE. (**e**) Technical illustration of daytime in MARTE. (**f**) Technical configuration in MARTE in the interphase between daytime and nighttime. (**g**) Technical illustration of nighttime in MARTE.

The purpose of the water cycle simulation in the MARTE chamber is to favor the survival of the biological sample. Experimentally, we recreated the environmental daytime/nighttime variations that correspond to the SOL in Mars (a Martian day, similar to Earth with 24 h and 37 min) as shown in Table 3 and in Figure 9, in which the daytime period is a quarter of the nighttime period.

Figure 9 illustrates a graphical comparison between the hydrological cycle of the red planet, mainly focused on the water condensation and evaporation and the solutions adopted to recreate this cycle in the MARTE vacuum chamber.

In MARTE, in the daytime, there is mainly condensation and evaporation while, in the nighttime, freezing and sublimation. In the special case of the interphase between daytime and nighttime, there is evaporation and freezing and in the interphase between the nighttime and daytime, melting, and evaporation.

Initially, the biological sample collected was stored in a climatic chamber at ambient pressure that reproduces the daytime/nighttime cycle and the temperature remained constant at 20 °C until its use. The experiment in MARTE started with the pressure at 20 mbar of CO_2_ and the temperature in the sample holder at 10 °C with the radiation source on (Figure 9c,e). The maximum radiation was reached 15 min after the source was turned on, which we can interpret as a sunrise. After 6 h, we have the interphase period (Figure 9f). In the interface between daytime and nighttime, there is a sharp decrease in pressure down to 1 mbar that produces a rapid freezing of the sample from top to bottom at −20 °C, which is maintained with the cryostat at a temperature of −15 °C that simulates nighttime on the red planet (Figure 9g). Although on Mars, night times are much colder, they often reach −80 °C, and occasionally even colder temperatures.

In MARTE, we used two methods to cool the sample. We cooled the sample from below by extracting heat with the sample holder with a glycol circuit and from above by absorption when pumping the vacuum chamber at maximum speed. Thus, we created a layer of superficial ice that protects the sample against external radiation and minimizes the loss of humidity due to evaporation.

Radiation control is carried out by turning the Xenon source on and off. Relative humidity is rapidly checked with the temperature decrease in the nighttime cycle. The appearance of ice by absorption and the constant temperature of −15 °C minimizes relative humidity up to the threshold value (<10%) at 20 mbar pressure on MARTE. In the 6-h daytime cycle, at dawn (Figure 9e), the sample holder temperature was increased up to 10 °C with a ratio of 1 °C/min and the Xenon source was turned on. As the temperature rose, the ice melted (the hydration sensor indicates that the sample thaws smoothly, decreasing its resistance), and the water started evaporating. This is the point where we begin to recreate through water injections the condensation that mimics the minimal hydration and relative humidity conditions that favor the sample survival.

**Figure 10:**
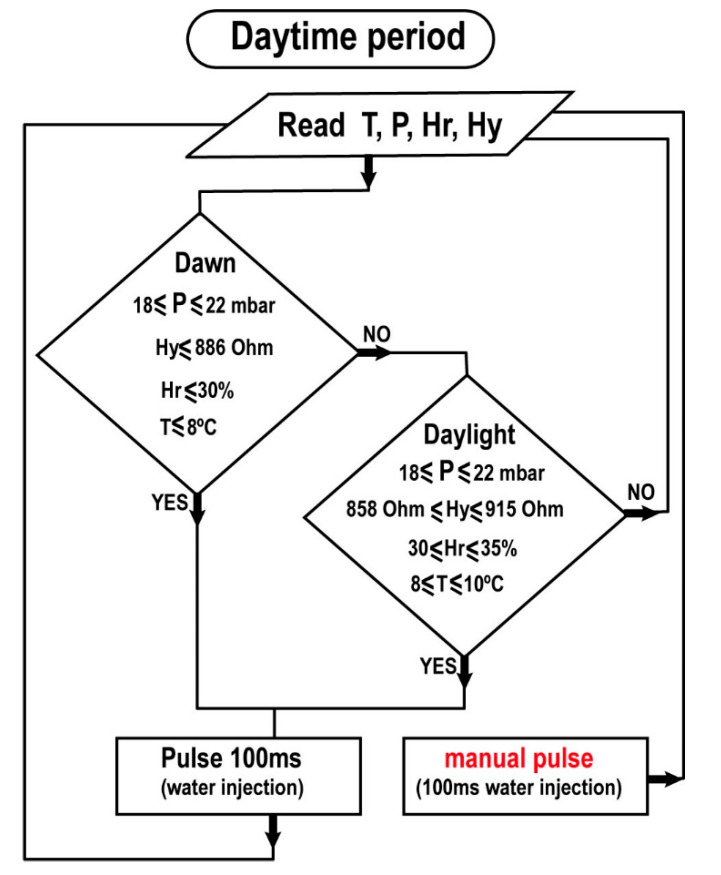
Injection algorithm during the daytime period. The variables are pressure P, sample temperature T, relative humidity Hr, and hydration Hy.

We designed a homemade software with LabVIEW^®^ (Figure 10) to recreate the condensation inside the MARTE vacuum system by means of water pulse injections. The injections are produced considering the values of pressure P, sample temperature T, relative humidity Hr, and hydration Hy. The system automatically injects water pulses based on the relation of these variables. In all cases, the pulses are 100 ms.

This injection condition relates the four environmental variables in the MARTE daytime cycle considering uncertainty and measurement system error. In daytime, we have two periods, dawn, and daylight. At dawn, the sample is thawing. It is particularly important not to over-wet the sample (synchronize the thawing sample with the pulse injection taking into account the hydration measurements) not to excessively increase the humidity and hydration of the sample. Thawing should be allowed to occur by keeping the sample moist. After the defrosting period is over, it is in the maintenance phase in daylight. In these periods, the sample holder temperature is maintained at 10 °C.

## 4. Results

We conducted two simulations for a period of five SOLs. In the first simulation, we introduced a moss sample in optimal conditions. The second simulation was with the same sample but sterilized in an autoclave after the first simulation period ended. In this way, we have a reference of the same sample in which we know that photosynthesis will not occur. We chose this method to verify by means of photographs, mass spectrometry, and environmental sensors (T, P, Hr, Hy) the survival of the biological sample under extreme conditions in the MARTE chamber.

The biologically active sample (green moss) is called Z (zeta) and the sterilized sample is called 0 (zero).

**Figure 11:**
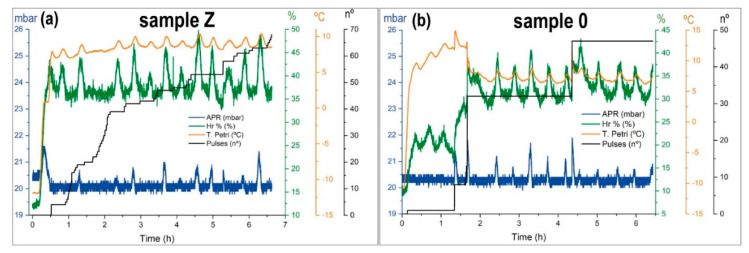
Environmental variables during the third SOL. Pressure (mbar) is in blue, relative humidity (%) in green, the sample temperature (°C) in orange. The number of pulses injected in black. (**a**) Sample Z. (**b**) sample 0.

For sample Z, the experiment started with the sample hydrated at room temperature and atmospheric pressure with a mass of 97.31 g. As seen in Table 4, in the first SOL, there was no injection, the sample had good hydration, and began to dry up. The effect of low pressure and temperature caused water evaporation. From the second SOL, the system started to inject water pulse to ensure the sample and maintain the environmental variables in the range. In Figure 11a, we show the third SOL as representative of all series. At dawn, the sample began to thaw; consequently, the environmental variables increased the values. Only the pressure increased above the expected. The rate of evaporation was higher than the pumping speed. The sample retained water inside, and the hydration was in the range for Martian simulation. It is worth noting the oscillations of temperature and relative humidity. These oscillations are related to the control of the pressure in dynamic mode and the temperature of the sample holder, due to the thermal gradient between the temperature of the glycol conducts inside MARTE (−15 °C) and the set point temperature of the sample holder at 10 °C (Figure 11). A minimal variation of the sample holder temperature causes a change in the evaporation rate.

Due to this evaporation, the sample temperature ranges are between 10 and 7 °C. In the case of the pressure (in blue) in Figure 11, the fluctuations depend on the water evaporation with the pulse injections, and the CO_2_ pressure control. Sample Z evaporated to its maximum and maintained this level for the rest of the simulation. The behavior of the sample was very stable in all variables within the ranges set by the software. At the end of the experiment, the sample had lost 14.3 g and consumed 71.72 g. It lost water in a controlled manner in the hydration simulation but maintained good conditions of relative humidity.

The simulation experiment for the 0 or sterilized sample is shown in Table 5 and in Figure 11b as representative of all series. Before introducing the sample in MARTE, this is manually hydrated with approximately the same mass as sample Z. In this first SOL there were no injections, the sample rapidly lost hydration. In the second SOL, the sample was within the limits of hydration. Injections were conducted and relative humidity fluctuated with the thermal oscillations of the sample holder. In the third SOL, after dawn, the sample was completely dry. The nighttime period with freezing by absorption was insufficient to retain the sample humidity. Only with manual injections was it possible to maintain a good humidity level. In SOL 3 (Figure 11b) and SOL 5, water injection was performed manually to try to start the hydration cycle (Table 5). The sample did not have a humidity retention mechanism and dried quickly in low pressure. In the fourth SOL, the system evolved naturally (without injections) and the sample was completely dry. The relative humidity dropped to the threshold pressure level at 20 mbar. In the absence of water there was no evaporation, and the sample temperature was more elevated than when the sample was hydrated. The sample temperature rose to 15 °C. In sample 0, the water loss was 30.48 g and the total water injected was 32.12 g.

**Figure 12:**
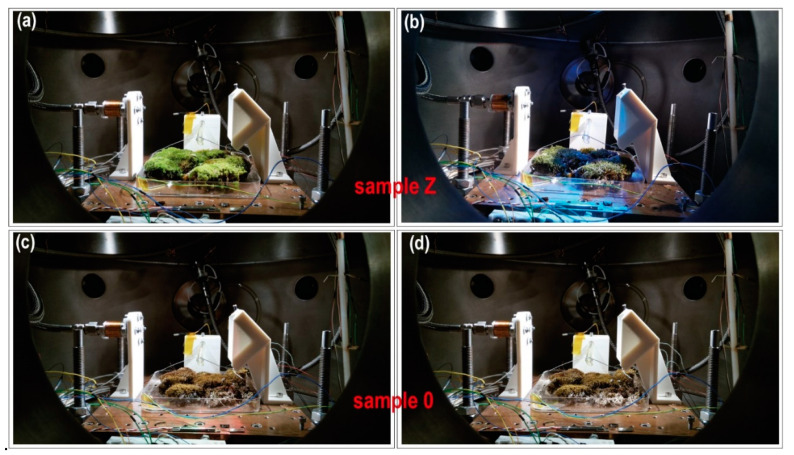
Photographs of the moss sample. (**a**) Photograph of sample Z before the simulation. (**b**) Photograph of sample Z after SOL 5. (**c**) Photograph of sample 0 before the simulation. (**d**) Photograph of sample 0 after SOL 5.

Figure 12 shows the initial and final appearance for both samples. A large difference in appearance (color and brightness) of sample Z was observed in the first SOL (Figure 12a) when compared to the last SOL (Figure 12b). However, for sample 0, there were no differences in color and brightness between the hydrated sample (Figure 12c) and the completely dry sample (Figure 12d) after the fifth SOL. Figure 12d shows the background of the sample with a whitish hue, which denotes that it is very dry.

**Figure 13:**
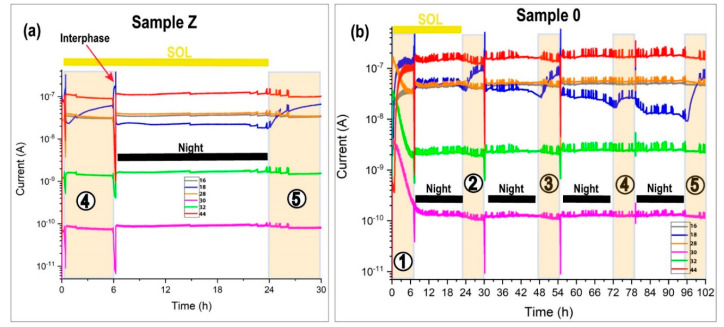
MID of samples Z and 0. The shaded areas correspond to daytime. The ions (q/m) 16, 18, 28, 30, 32, and 44 correspond mainly to oxygen, water, nitrogen, nitrogen dioxide, and carbon dioxide. The numbers correspond to the SOL number. The interphase is the region between de daytime and the nighttime. (**a**) MID of sample Z from the fourth dawn. (**b**) Complete MID of sample 0.

Figure 13a shows the MID of the last two daytime periods of sample Z and Figure 13b reveals the complete MID of sample 0. In the MID, it is possible to view the transition effect in the interphase at sundown and dawn and the pulse effect for all mass. At dawn, the mass 18 (water) increased due to defrosting and evaporation. The xenon source was on and the sample holder temperature was 10 °C. In the daytime, we mimicked the condensation effect with the water pulse injection hydrating the sample. This process undergoes an abrupt change at the end of the daylight at sundown, in which MARTE was pumped to the maximum speed; closing the CO_2_ inlet expecting to leave the chamber at one mbar. In this process, water predominates (mass 18). It is the predominant mass inside the vacuum equipment of the RGA (10^−5^ mbar). Water is adsorbed on the walls of the vacuum chamber. With a temperature of −20 °C in the sample, the base pressure quickly increased to 20 mbar and brought on nighttime. The gas composition atmosphere changed quickly, reproducing the atmosphere of the red planet with 95% CO_2_. The ion predominated with a mass of 16 over 18 due to the greater contribution of carbon dioxide in the fractionation within the quadrupole. In the nighttime period with the sample frozen and without ultraviolet radiation, the biological sample entered the photo-reparation phase. The moss surface layer acts as a protective layer for the rest of the sample.

In sample Z, the mass evolution was constant in the nighttime and there were only changes in the daytime due to pulse injections. The sample had a good repeatability in the five SOLs.

The general appearance of both graphs was the same, although there were two differences. The pressure control through the RME valve acted less on sample Z than on sample 0 to maintain 10^−5^ mbar in the RGA. The MID of sample Z had less peaks due to pressure variations. The second difference is in the intensity of mass 18. The humidity is less in sample 0 compared to sample Z. The sterilized sample did not demand water with the same injection conditions.

## 5. Discussion

For the biological sample, the main goal of the water injection pulses was to keep the moss green inside MARTE. We needed a combination of humidity, hydration, and UV plus optical radiation over the sample. Pressure, the gas atmosphere composition, and temperature are the limits of the simulation when mimicking the water cycle. The Xenon source mimicking solar radiation in the visible region, though the UV radiation, is low when compared to Martian radiation. To simulate the radiation effect in the UV region it would be necessary to increase the number of SOLs. In our simulation, the damage for UV radiation is low for a few SOLs. It is especially important if the global objective is the radiation damage study, to increase the radiation dose.

Most of the water drops fell by decantation on the Petri plate and were absorbed by the sample. Water not absorbed by the sample evaporated directly from the sample surface (droplets) and helped increase relative humidity. For this reason, even despite the high number of pulses, the sample ended the experiment with a high level of relative humidity and low hydration compared to the initial conditions of the sample on Earth. Sample Z retained water and enabled more controlled evaporation. During the daytime, the process of photosynthesis needs water. This makes the system more hydrated and more stable in the vacuum chamber. In addition, the relative humidity level (mass 18) remained higher in sample Z with the green moss. However, in the case of the 0 or sterilized sample, there was no water retention, so the system had to be constantly adjusted.

Although the hydration conditions are the same, in sample Z, there were twice as many injections as in sample 0 because the green moss needed more water during the daytime. The MID was very stable throughout the experiment. The automatic injections respond to the need of the sample to consume liquid water to maintain the moss in the best humidity and hydration conditions (Table 4). A much smaller number of oscillations in the RGA was observed in pressure regulation compared to sample 0 (Figure 13). However, in the 0 sample, a vastly different behavior was observed. Manual injections of day 3 and 5 did not suffice to keep the humidity and the hydration self-supporting in the daytime. The sample did not retain water and evaporated easily. On day 4 without pulses, the water was maintained although it had a slight rebound in the daytime due to the increase in temperature. The sample continuously degassed. In the daylight with the sample holder at 10 °C without water injections (Figure 13b at daytime 4), the mass 18 increases due to sunrise and then remains unchanged, indicating that it continues to degas. The general tendency for the sample was to dry up. The sterilized sample lacked the necessary regulatory mechanisms to retain water inside. All the injected water is pumped without being absorbed by the sterilized sample.

In the MID, molecular oxygen ion-mass 32 is desirable as an indicator of oxygen production due to photosynthesis since ion-mass 16, also oxygen, has contributions from CO_2_ and H_2_O inside the quadrupole mass spectrometer. Mass 32 is possible for two contributions. The first one due to the residual oxygen of the vacuum chamber (cavities and holes). This is very well observed at the beginning of the experiment since mass 32 decreased until all the air (nitrogen and oxygen) in the vacuum chamber was replaced by CO_2_ and H_2_O. The second and expected contribution is due to photosynthesis by the green moss inside MARTE.

During the daytime, there is no partial increase in mass 32 (molecular oxygen) with respect to the nighttime that allows the contribution due to photosynthesis to be established conclusively. However, the study of the humidity and hydration conditions of water pulse injections together with the photographs of the biological sample (Figure 12b) are a good indication that the sample stayed in the right conditions to produce oxygen by photosynthesis if we consider the green sample as biologically active. Furthermore, we checked the stability of sample Z through the MID under the most favorable hydration conditions to keep the sample green.

The effect of freezing the sample from top to bottom is like what occurs on Earth. We created one protective ice-water layer to minimize the outgassing (loss humidity due to water evaporation), which retained the water inside the holes in the biological sample and created a protective layer for UV radiation. This layer disappeared at dawn and enabled the hydration of the sample to decrease progressively in vacuum.

## 6. Conclusions

We expanded the possibilities of the MARTE chamber by turning it into a climatological chamber beyond the simulation of a valid planetary atmosphere to test environmental instrumentation for Mars’ atmosphere and surface. MARTE is now a platform capable of recreating the environmental variables related to the evolution of water in a spatial environment. Pressure, temperature, radiation, relative humidity, and hydration enable us to generate processes of condensation, evaporation, freezing, melting, and sublimation that are postulated as keys to simulate the possible niches of life in spatial environments. We validated our experimental development by using a biological sample from a glacial environment that has allowed us to design and optimize the appropriate tools to maintain a daytime/nighttime cycle, which enabled us in turn to study survival in real time through photographs, and the study of gases inside a vacuum chamber. Moss adapts in the Martian environment and feeds back from the hydrological cycle. The increase of mass 32 in the periods of solar radiation in daytime with respect to nighttime is not the definitive method of verification of oxygen production since it depends on the type and the sample surface and of the vacuum volume in the simulation chamber. Taking photographs is a good indication to estimate survival (colors). Currently, MARTE is a unique tool to recreate extreme water environments in a vacuum and thus to study the evolution of biological and geological samples as can happen on the surface of planets, such as Mars.

In MARTE, we used two methods to cool the sample. We cooled the sample from below by extracting heat with the sample holder with a glycol circuit and from above by absorption when pumping the vacuum chamber at maximum speed. Thus, we created a layer of superficial ice that protects the sample against external radiation and minimizes the loss of humidity due to evaporation. This will allow simulation of the interaction between water and ice at the interphase on a possible case scenario on Mars sunset and sunrise, and its interaction with different samples of brines and biological samples, such as cyanobacteria.

The ALI technique ensures the existence of liquids in vacuum and enables the simulation of the hydrological cycle inside the MARTE chamber. Water injection in the form of droplets on the surface and in drops at the bottom of the sample keep the relative humidity, temperature, and hydration at suitable values for the survival of a biological sample in an atmosphere of 20 mbar of CO_2_ as can happen on the red planet.

The injection of liquids in a vacuum within a climatic chamber is a powerful tool to simulate habitability and geochemical processes in environments of astrobiological interest.

From the biological point of view, in the near future, the MARTE chamber will have more direct methods in vacuum, such as a pulse-amplitude-modulated (PAM) chlorophyll fluorimeter to measure chlorophyll fluorescence on biological samples, and outside of vacuum, the RNA (ribonucleic acid) extraction is required to perform a definitive assessment to ensure the sample survives.

From the geological point of view, it is interesting to note the simulation with the liquid injection system to create the evolution of a brines torrent similar to those found on the slopes of the red planet.

## Figures and Tables

**Table 1:** Environmental variables in MARTE and in the red planet [15].

	MARTE	Mars
Composition of gases	100% CO_2_	95% CO_2_, 2.7% N_2_; 1.6% Ar; 0.13% H_2_0; 0.08% CO
Pressure	1–20 mbar	~8 mbar
Temperature	253 K < T < 283 K	170 K < T < 283 K (Summer)
Relative humidity	10% < Hr < 35%	<35% max. (Summer)
Radiation	Xenon, 666 W/m^2^	Sun, 550 W/m^2^

**Table 2:** Spectral range and Xenon irradiance by the UV photodiodes used in the “RadSensor”.

Photodiode	Spectral Range (nm)	Xenon Irradiance (W/m^2^)
UVA	320–395	1.46
UVB	265–322	0.33
UVC	225–280	0.25

**Table 3:** MARTE’s range of environmental variables during hydrological cycle experiments.

MARTE	Daytime	Interphase	Nighttime
Pressure (mbar)	20	1	20
Temperature (°C)	10	−20	−15
Radiation (%)	100	-----	0
Relativity Humidity (%)	35	-----	10

**Table 4:** Summary of the injection process in the Z sample.

SOL	Injections	Mass Water Injected (g)	Sample Mass (g)
1	0	0	97.31
2	96	21.12	
3	68	14.96	
4	84	18.48	
5	78	17.16	83.01

**Table 5:** Summary of the injection process in the 0 sample.

SOL	Injections	Mass Water Injected (g)	Sample Mass (g)
1	0	0	98.51
2	48	10.56	
3 ^1^	47	10.34	
4	0	0	
5 ^1^	51	11.22	68.03

^1^ SOLs with manual pulses.

## Supplementary Materials

The following are available online at https://www.mdpi.com/1424-8220/20/21/6150/s1, Video S1: Dewsensor. Video taken during the injection pulse calibration.
